# Molecular evidence of two cryptic species of *Stramonita* (Mollusca, Muricidae) in the southeastern Atlantic coast of Brazil

**DOI:** 10.1590/1678-4685-GMB-2015-0199

**Published:** 2016-07-07

**Authors:** Juliana Beltramin De Biasi, Acácio Ribeiro Gomes Tomás, Alexandre Wagner Silva Hilsdorf

**Affiliations:** 1 Núcleo Integrado de Biotecnologia, Universidade de Mogi das Cruzes, Mogi das Cruzes, SP, Brazil; 2 Centro Avançado de Pesquisa Tecnológica do Agronegócio do Pescado Marinho, Instituto de Pesca, Santos, SP, Brazil

**Keywords:** COI, 16s rRNA, mitochondrial DNA, southern oyster drill, Brazilian coast

## Abstract

Snails of the genus *Stramonita* are commonly found in the rocky intertidal habitat of the western Atlantic Ocean coast. They belong to a monophyletic taxon that occurs along the tropical and warm-temperate Atlantic and eastern Pacific rocky shores. This genus comprises different valid species and members of the *S. haemastoma* complex. In the present study, samples of *Stramonita* were collected from three different regions of southeastern Brazil. Partial sequences of two mitochondrial genes, COI and 16S rRNA, were used to compare nucleotides sequences between *Stramonita* specimens. Levels of nucleotide divergence greater than 2% across the three sampled regions were used for differentiation at the species level. One of the identified species was *S. brasiliensis*, which has recently been described by molecular analysis; the other species may represent *S. haemastoma,* not yet described in the southeastern Brazilian coast.

In marine environments, genetically different organisms may arise due to ecological, geological, and oceanographic barriers under either allopatric, parapatric or even sympatric models of evolution (Rocha *et al.*, 2005; Von der Heyden *et al.*, 2011). Cryptic species indistinguishable by morphological criteria are often classified within a single taxon (Bickford *et al.*, 2007). The existence of cryptic species seems to be common in marine organisms, such as in Sciaenidae fishes (Vinson *et al.*, 2004; Santos *et al.*, 2006), as well as in numerous invertebrates (Thorpe *et al.*, 2000), including Penaeid shrimp (Gusmão *et al.*, 2005). Although not regarded as cryptic species, some species of the genus *Stramonita* contain features which make identification challenging; these features are characterized by a wide range of ecophenotypic variations, which can be corroborated by the morphology and coloration variability of the shell due to environmental factors (Butler, 1985; Houart and Gofas, 2010). As a result, these species have been misidentified, which has led to taxonomic controversy (Liu *et al.*, 1991; Vermeij, 2001).

*Stramonita*, also known as southern oyster drill, is a predatory marine gastropod mollusc found along rocky intertidal habitats of the Atlantic and eastern Pacific Oceans (Ramírez *et al.*, 2009). Due to variation in shell shape and coloration, the two *S. haemastoma* and *S. rustica* are divided into three subspecies: *Stramonita haemastoma floridana* in the Caribbean, *Stramonita haemastoma canaliculata* in the Gulf of Mexico, and *Stramonita rustica bicarinata* on the South Atlantic islands (Clench, 1947; Butler, 1985; Harding and Harasewych, 2007).

Mitochondrial gene sequences have been used in molecular taxonomy studies of *diverse* marine life forms (Knowlton, 2000; Bucklin *et al.*, 2011; Trivedi *et al.*, 2015). Molecular characterization using mitochondrial DNA has been carried out for different gastropod group taxa (Donald *et al.*, 2005; Pfenninger *et al.*, 2006; Perez and Minton, 2008; Kool and Galindo, 2014).

Here, we evaluated individuals of the genus *Stramonita* sharing the same rocky intertidal habitats, assuming that: (i) both putative species show subtle morphological differentiation; (ii) there is no report of the presence of two species of *Stramonita* along the southern coastline of Brazil; (iii) the sustainable use of this marine resource under exploitation by coastal fishing communities depends on accurate species identification. Therefore, in the present work we aimed to assess the likely presence of two sympatric species, and to discuss the implications of the outcomes for conservation of these ecologically important intertidal marine snails.

Twenty individuals of *Stramonita* were randomly sampled at three regions along the State of São Paulo coast, Brazil: Ilha Bela (23°48'S, 45°21'W), Santos (23°59'S, 46°18'W), and Peruíbe (24°21' S, 46°60'W). The identification of putative species was performed using subtle morphological variations based on Clench (1947), Rios (1994), and Matthews (1968). DNA was extracted from the foot muscle using the IlustraTM Tissue & Cells genomic Prep Mini Spin Kit (GE Healthcare Life Sciences, Buckinghamshire, UK) following the manufacturer's instructions. The partial region of the genes 16S ribosomal RNA (or 16S rRNA) and *cytochrome c oxidase* subunit I (or COI) were amplified using PCR. The primer pairs used for the 16S rRNA gene amplification were: 16Sar-L (5-CGCCTGTTTAACAAAAACAT-3) and 16Sbr-H (5-CCGGTTTGAACTCAGATCACGT-3) (Palumbi, 1996). The primers used for the amplification of the COI gene were: dgLCO1490 (5- GGTCAACAAATCATAAA GAYATYGG-3) and dlHCO2198 (5- TAAACTTCAGGG TGACCAAARAAYCA-3) (Meyer, 2003). PCR amplifications were performed in a final volume of 50 μL. Each reaction contained a buffer of PCR 1 X, 100 mMdNTPs, 2.5 mM of MgCl2, 0.5 μM of each primer, 1 U/μL *Taq* DNA Polymerase (Invitrogen™, Carlsbad, USA), deionized water, and 50 ng/μL of genomic DNA. The thermal regime for the 16S rRNA consisted of an initial denaturalization to 94 °C for 1 min, followed by 35 cycles at 94 °C for 30 sec, 52 °C for 30 sec, and 72 °C for 1 min, with a final extension at 72 °C for 10 min. PCR conditions for the COI gene included initial denaturalization at 94°C for 1 min, followed by 30 cycles at 94 °C for 30 s, 45 °C for 40 s, and 72 °C for 1 min, with a final extension at 72 °C for 10 min. PCR products were purified and sequenced in an automatic ABI 3700 sequencer (PE Applied Biosystems, Foster City, CA).

All sequences were confirmed by sequencing both strands. The consensus sequence for the two strands was obtained using CodonCode Aligner v.2.0.4 software (CodonCode Corporation, Dedham, Massachusetts, USA). Sequences were aligned using the Clustal W interface with the Mega 5 software (Tamura *et al.*, 2011). The saturation levels of the molecular data were determined using DAMBE software version 5.0.39 (Xia and Xie, 2001). Transition and transversion were plotted based on the TN93 model from each gene data set (Tamura and Nei, 1993). Distance estimates of genetic divergence (p-distance) over sequence pairs, between and within groups, were conducted with MEGA 5. The phylogenetic trees were built by Maximum Likelihood (ML) carried out with MEGA 5, based on alignments of concatenated COI and 16S rRNA genes. Modeltest 3.7 (Posada and Crandall, 1998) was applied to find the best fitted model for ML. The consistency of topologies was measured by the bootstrap method (1000 replicates) and only confidence values > 50% were reported. To compare the *Stramonita* species sequences achieved herein with the sequences of *Stramonita* type locality worldwide, we used COI and 16S sequences of *S. haemastoma* species from Tenerife, Spain (FR695793.1, COI and HE584302.1, 16S), *S. floridana* from Florida, USA (FR695848.1, COI and HE584301.1, 16S), *S. brasiliensis* from Ilha Bela, São Paulo, Brazil (FR695844.1, COI and HE584298.1, 16S), and *S. rustica* from Brazil and Costa Rica (FR695847.1, COI and HE584303.1, 16S).

Forty forward-reverse new partial sequences from 20 specimens were obtained, with a total of 610 bp for the COI gene sequence alignment with nucleotide frequencies and 451 bp for the 16S rRNA gene sequence. The conchological variation of *Stramonita* was not taxonomically informative for species identification (Figure 1). The best evolutionary model was the HKY+G (Hasegawa, Kishino and Yano + invariable sites). Analysis of substitutions (transitions and transversions) showed no saturation for either of the two genes. The ML concatenated tree generated from the COI and 16S rRNA gene sequences clustered the *Stramonita* subspecies into three distinct clades (Figure 2).

**Figure 1:**
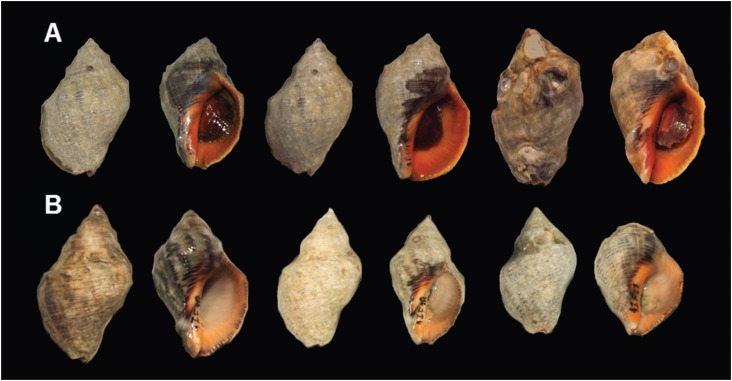
Color variation and shape of shells of *Stramonita* cf. *haemastoma* (A) and *S. brasiliensis* (B).

**Figure 2:**
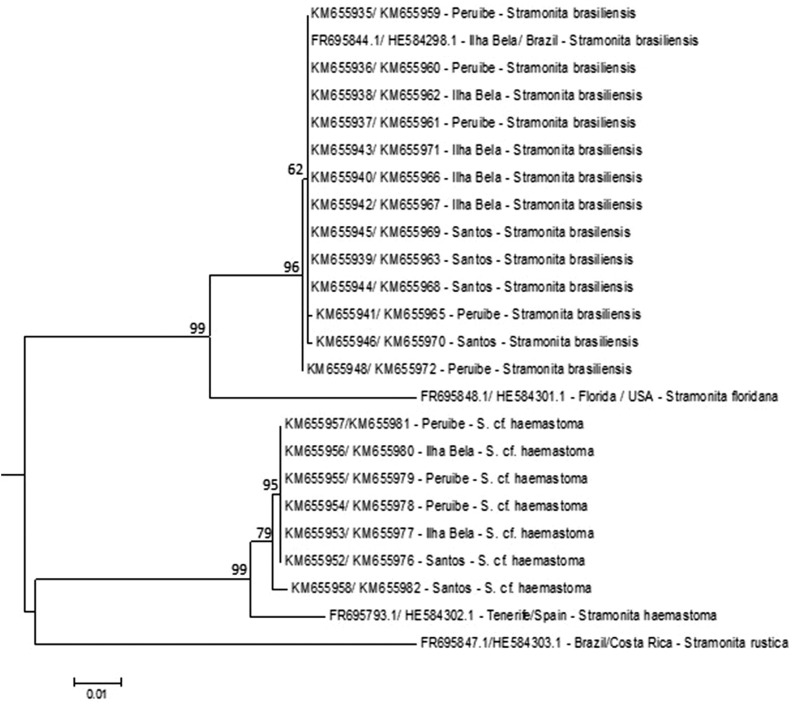
Maximum Likelihood tree as a result of concatenated data sets of COI and 16S rRNA gene sequences of the *Stramonita haemastoma* complex. Branches are supported by bootstrap values above 50%. GenBank access numbers and location are shown in Table 1.

**Table 1:** Sampling locations, geographic coordinates, Genbank access number, and morphological identification of the *Stramonita* individuals.

Location	COI	16S	Coordinates	Morphological identification	Molecular identification
	GenBank access n.	GenBank access n.			
**Peruíbe**	KM655935	KM655959	24°19'2″ S 46°59'44″W	*S. floridana*	*S. brasiliensis*
	KM655936	KM655960		*S. floridana*	*S. brasiliensis*
	KM655937	KM655961		*S. haemastoma*	*S. brasiliensis*
	KM655941	KM655965		*S. floridana*	*S. brasiliensis*
	KM655948	KM655972		*S. haemastoma*	*S. brasiliensis*
	KM655954	KM655978		*S. haemastoma*	*S.* cf. *haemastoma*
	KM655955	KM655979		*S. haemastoma*	*S.* cf. *haemastoma*
	KM655957	KM655981		*S. haemastoma*	*S.* cf. *haemastoma*
**Ilha Bela**	KM655938	KM655962	23°46'28″S 45°21'20″W	*S. floridana*	*S. brasiliensis*
	KM655940	KM655966		*S. haemastoma*	*S. brasiliensis*
	KM655942	KM655967		*S. floridana*	*S. brasiliensis*
	KM655943	KM655971		*S. floridana*	*S. brasiliensis*
	KM655953	KM655977		*S. haemastoma*	*S.* cf. *haemastoma*
	KM655956	KM655980		*S. haemastoma*	*S.* cf. *haemastoma*
**Santos**	KM655939	KM655963	23°57'52″S 46°20'0″W	*S. floridana*	*S. brasiliensis*
	KM655944	KM655968		*S. haemastoma*	*S. brasiliensis*
	KM655945	KM655969		*S. floridana*	*S. brasiliensis*
	KM655946	KM655970		*S. floridana*	*S. brasiliensis*
	KM655952	KM655976		*S. haemastoma*	*S.* cf. *haemastoma*
	KM655958	KM655982		*S. haemastoma*	*S.* cf. *haemastoma*

There was no genetic distance within clades for *S. brasiliensis* and for *S.* cf. *hemastoma* collected for this study. The mean genetic divergence between species were: 8% (*S. brasiliensis* and *S. haemastoma*), 10% (*S. brasiliensis* and *S. rustica*), and 9% (*S. haemastoma* x *S. rustica*). *S. floridana* (type locality: St Augustine Inlet, St James Co., Florida) diverged genetically from *S. brasiliensis* studied herein by 5%. At the same time, *S. haemastoma* (type locality: Tenerife, Canary Islands) diverged by 2% from *S.* cf. *haemastoma*.

In this study, molecular analyses have enabled the detection of strong differences between *Stramonita* individuals found on the Southeast coast of Brazil, indicating the existence of cryptic variation that is associated with the existence of different species of *Stramonita*. As reported by Clench (1947), the shells' morphological variations suggest an enormous ambiguity that indicates marked overlay. The reason is that certain environmental factors that intertidal gastropods are exposed to hamper the use of its external morphology for taxonomic identification (Liu *et al.*, 1991; Kool, 1993; Vermeij 2001; Claremont *et al.*, 2011). The two mitochondrial genes used in this study were effective in distinguishing both species as taxonomically distinct units with large genetic distances. A review in the literature about threshold values for molluscs species differentiation shows values between 1.9% and 14% (Mikkelsen *et al.*, 2007). Layton *et al.* (2014), analyzing the COI gene, used the threshold of 2% to delimitate species of molluscs. Claremont *et al.* (2011), working with *Stramonita,* obtained pairwise distances within species of 1.9% for *S. rustica*, 1.8% for *S. haemastoma*, 1.3% for *S. floridana*, and 0.8% for *S. brasiliensis*, and sequence divergence among species ranging from 6.8% to 12.0%. Other genetic and molecular sequences comparison approaches have been used in different studies to identify the occurrence of two sympatric and genetically distinct groups of *S. haemastoma* in Brazil (Udelsmann, 2009, Master of Science Thesis, UNICAMP, Campinas, Brazil). Other methods were used as well to report for the first time the presence of *Stramonita haemastoma floridana* in the Chesapeake Bay along the southeastern coast of the United States, as a result of isolated introductions or of northward expansions of this species (Harding and Harasewych, 2007).

Our results corroborate the findings of Claremont *et al.* (2011), which confirm the occurrence of the *S. brasiliensis* along the Brazilian coast. Claremont *et al.* (2011) also reported that *S. floridana* only inhabits the south Atlantic region of the United States and suggested that the *S. rustica* species occurs with *S. brasiliensis* along the Brazilian coast. In the present work, we have not verified the presence of *S. rustica*, which is extensively found along the Northeast coast of Brazil (Camillo *et al.*, 2004; Castro *et al.*, 2004). Regarding *S. haemastoma*, our results showed a genetic distance of 2% between the *S. haemastoma* from type locality of Tenerife - Spain, clustered with the samples collected for this work. Claremont *et al.* (2011) pointed out that shells from Brazil have generally been identified as *S. haemastoma*. These authors, having verified the original descriptions of all names listed as *Stramonita* species, believe that this species has still to be described. Surprisingly, although these authors collected their samples at the same site as we did our samplings, they did not find specimens other than *S. brasiliensis*. It is still premature to assert that *Stramonita* specimens occuring sympatrically with *S. brasiliensis* would be a new species, but S. haemastoma is considered a species complex, and the genetic distance between *S. haematoma* from Terefine/Spain and from our samples is at the borderline of species delimitation with COI sequence data (2%). It is important to point out that the limit of species resolution depends on the model of sequence evolution used. Different studies using COI on gastropod species relied on various models, such as Kimura 2-parameter (K2P) distances (Kool and Galindo, 2014; Layton *et al.*, 2014), Generalized time reversible model (GTR+I+Γ) (Pfenninger *et al.*, 2006), and Hasegawa, Kishino and Yano model (HKY+I+G) (Claremont *et al.*, 2011). Therefore, we would provisionally identify this species as *S.* cf. *haemastoma* until further analysis, such as sequence comparison with other genes, cytogenetic studies, internal anatomy, and marginal crenulation evaluations, be carried out for complete taxonomy determination of this taxon.

Finally, *Stramonita* species represent a food source and their commercial value is potentially high (Manzoni and Lacava, 2010). Therefore, the fishing exploitation of these intertidal marine molluscs can become a profitable business for small-scale coastal fishermen. The challenges in species recognition during fishing can create a serious depauperation, jeopardizing natural populations in *Stramonita* species. The molecular identification of two independent sympatric taxa cohabiting the same rocky shorelines is an important step for a sustainable management of these species, as their indiscriminate removal for commercial purposes could imperil the ecological balance of the intertidal rocky marine environment.
